# Prosthesis–Patient Mismatch Following Aortic Valve Replacement—A Comprehensive Review

**DOI:** 10.3390/jcm14248868

**Published:** 2025-12-15

**Authors:** Sriharsha Talapaneni, Danial Ahmad, Meghna Khandelwal, Monica Mesiha, Pooya Jalali, Nafiye Busra Celik, Sair Ahmad Tabraiz, Sedem Dankwa, Irbaz Hameed, Rita Milewski, Prashanth Vallabhajosyula

**Affiliations:** Division of Cardiac Surgery, Department of Surgery, Yale University School of Medicine, New Haven, CT 06510, USA; sriharsha.talapaneni@yale.edu (S.T.); danial.ahmad@yale.edu (D.A.); meghnakhandelwal99@gmail.com (M.K.); monica.mesiha@med.cuny.edu (M.M.); pooya.jalali1995@gmail.com (P.J.); nafiyebusra.celik@yale.edu (N.B.C.); tabraiz.sairahmad@mayo.edu (S.A.T.); sedem.dankwa@yale.edu (S.D.); irbaz.hameed@yale.edu (I.H.); rita.milewski@yale.edu (R.M.)

**Keywords:** patient prosthesis mismatch (PPM), aortic stenosis, aortic valve replacement

## Abstract

**Objective:** Prosthesis–patient mismatch (PPM) occurs after aortic valve replacement (AVR) when the effective orifice area of the implanted prosthetic valve is small relative to the patient’s body surface area. Beyond simply elevating transvalvular gradient, PPM profoundly affects cardiac remodeling, coronary physiology, and ultimately patient survival. This comprehensive review synthesizes current evidence regarding PPM pathophysiology, clinical consequences, and therapeutic strategies. **Methods:** We conducted a narrative review of PPM in surgical (SAVR) and transcatheter (TAVR) aortic valve replacement. PubMed and Embase were systematically searched using terms related to AVR and PPM and reference lists of key studies and reviews were screened. Studies addressing PPM prevalence, hemodynamic impact, clinical outcomes, and mitigation strategies were included. **Results:** PPM, defined as an iEOA ≤ 0.85 cm^2^/m^2^ (moderate) or ≤0.65 cm^2^/m^2^ (severe), demonstrates variable prevalence across studies, typically ranging from 5 to 30% after SAVR and 2–35% after TAVR. It is associated with increased transvalvular gradients, reduced left ventricular mass regression, persistent coronary flow abnormalities, higher rates of heart failure, and both early and late mortality. Supra-annular self-expanding transcatheter aortic valve replacement (TAVR) devices and newer generation stentless or bovine pericardial surgical valves exhibit lower PPM rates than older stented or porcine valves. Valve-in-valve (ViV) TAVR and bioprosthetic valve fracture (BVF) can improve outcomes in failed surgical valves but are less effective in small annuli. TAVR-in-TAVR procedures are limited by anatomic and technical constraints, especially in maintaining coronary access and minimizing residual gradients. **Conclusions:** PPM remains a common and clinically consequential complication of AVR that compromises long-term outcomes. It is largely preventable through accurate preoperative imaging, valve sizing, and consideration of annular enlargement. Optimal outcomes require matching valve characteristics to individual patient anatomy and physiology. In an era of expanding TAVR use, preventing PPM during the index procedure is critical to optimizing survival and preserving future reintervention options.

## 1. Introduction

Prosthesis–patient mismatch (PPM) is a well-recognized hemodynamic complication following aortic valve replacement (AVR), particularly in individuals with a small aortic annulus. First described by Rahimtoola in 1978, PPM can be considered to be present when the effective prosthetic valve area after implantation is less than that of a normal human valve [1]. This phenomenon occurs when a structurally and functionally normal prosthesis fails to provide adequate orifice area in relation to the patient’s body surface area (BSA) and cardiac output demands. This discrepancy leads to higher than expected postoperative transvalvular pressure gradients (TPG), mimicking residual aortic stenosis [2].

The clinical significance of PPM extends beyond its immediate hemodynamic consequences, as it exemplifies a form of non-structural valve dysfunction (NSVD) [3]. Recent evidence suggests that it is associated with increased transvalvular gradients, reduced left ventricular mass regression, persistent coronary flow abnormalities, higher rates of heart failure, and both early and late mortality [4]. In contrast to structural valve deterioration, PPM-related flow impairment results from prosthetic undersizing rather than leaflet pathology. As Rahimtoola aptly described, “all prostheses—mechanical or bioprosthetic—have an in vitro effective orifice area (EOA) smaller than that of the native valve, and therefore all valve replacements can be considered to be ‘stenotic,’ even if they are ‘normal’” [1,3]. This concept was further illustrated by Pibarot and Dumesnil’s vivid analogy: PPM is akin to “implanting a mouse’s valve in an elephant’s aorta”, highlighting a situation in which the native stenotic valve is excised, but a residual outflow obstruction persists due to undersizing of the prosthesis [5].

Patients presenting with small aortic annuli face particular challenges in this context, as anatomical constraints may limit the size of the prosthesis that can be safely implanted, thereby predisposing to clinically significant PPM. This review provides a comprehensive analysis of PPM mechanisms, diagnostic criteria, clinical implications, and therapeutic strategies for optimization of patient outcomes.

## 2. Hemodynamic Principles

The underlying hemodynamic burden of PPM can be explained by the hydraulic equation, transvalvular pressure gradient (TPG) = Q^2^/(k × EOA^2^), where Q represents transvalvular flow, and k is a constant [2]. This equation demonstrates the exponential relationship between effective orifice area (EOA) and pressure gradient, whereby even modest reductions in EOA result in disproportionate elevations in TPG, particularly in patients with higher BSA and correspondingly greater cardiac output requirements.

For example, assuming a normal cardiac index of 3 L/min/m^2^, a prosthesis with an EOA of 1.3 cm^2^ may result in a mean TPG of approximately 13 mmHg in a patient with a BSA of 1.5 m^2^. However, the same prosthesis in a patient with a BSA of 2.5 m^2^ could yield a TPG nearing 35 mmHg, with even more pronounced gradients during exertion [2]. Thus, is it important to consider indexed EOA (iEOA) and patient-specific characteristics during surgical planning to prevent clinically significant PPM [6,7]. Furthermore, these gradients may become substantially more pronounced during periods of increased cardiac output, such as exercise or physiological stress.

## 3. Diagnostic Parameters and Classification

The indexed effective orifice area (iEOA), defined as valve EOA divided by the patient’s BSA, has emerged as the most reliable metric to characterize PPM [5]. Among various echocardiographic and hemodynamic parameters, iEOA demonstrates the strongest correlation with postoperative transvalvular pressure gradients (TPG), providing a practical assessment tool for determining whether a prosthetic valve can adequately meet individual patient cardiac output demands [8].

The relationship between TPG and iEOA is curvilinear, with an exponential increase in gradients observed when the iEOA falls below 0.8–0.9 cm^2^/m^2^ [5]. Based on this relationship, an iEOA ≤ 0.85 cm^2^/m^2^ is generally accepted as the threshold for defining PPM in the aortic position [9]. PPM is classified as moderate when the iEOA is between 0.66 and 0.85 cm^2^/m^2^, and severe when it is ≤0.65 cm^2^/m^2^ [9].

In obese patients (body mass index [BMI] ≥ 30 kg/m^2^), the iEOA may overestimate the severity of PPM due to increased BSA relative to actual cardiac output [7,10]. As such, the Valve Academic Research Consortium 3 (VARC-3) recommends lower thresholds for diagnosing PPM in this population [11]. For obese patients moderate PPM is defined as an iEOA between 0.56 and 0.70 cm^2^/m^2^, and severe PPM as ≤0.55 cm^2^/m^2^ [5,10,12]. For comparison, in the mitral position, an iEOA ≤ 1.20 cm^2^/m^2^ is generally considered the threshold for PPM [12]. While these standardized thresholds provide valuable benchmarks for clinical assessment and research consistency, important limitations must be acknowledged. Applying standard iEOA cutoffs to obese patients may categorize valves as mismatched when they are actually hemodynamically adequate for the patient’s true physiological needs.

In clinical practice, these considerations necessitate a nuanced approach to PPM assessment. Rather than rigidly applying categorical thresholds, clinicians should interpret iEOA as a continuous hemodynamic variable, utilize obesity-adjusted VARC-3 criteria when BMI ≥ 30 kg/m^2^ (moderate PPM: iEOA 0.56–0.70 cm^2^/m^2^; severe PPM: ≤0.55 cm^2^/m^2^), and recognize that lower iEOA values confer progressively greater risk rather than discrete categories of harm. The primary clinical value of iEOA lies in preoperative planning, where predicted iEOA calculations enable proactive prevention of PPM through appropriate valve selection and consideration of annular enlargement techniques, particularly in patients with small annuli, where the risk is greatest [13,14].

## 4. Evaluation and Assessment of PPM

The potential for prosthesis–patient mismatch can be systematically evaluated preoperatively through multimodality imaging approaches, enabling proactive risk stratification and treatment planning [3]. This predictive assessment has become increasingly sophisticated with advances in imaging technology and represents a paradigm shift from reactive management to preventive strategies [9].

Cardiac-gated multidetector computed tomography (MDCT) has emerged as the cornerstone imaging modality for preoperative assessment, particularly in transcatheter aortic valve replacement (TAVR) candidacy evaluation [15,16]. MDCT provides precise three-dimensional measurements of critical anatomical parameters, including aortic annular area and perimeter, which are fundamental determinants of appropriate valve sizing [17]. Unlike surgical aortic valve replacement (SAVR), where prosthesis selection relies on intraoperative sizing techniques, TAVR prosthesis dimensions are predetermined based on native annular morphology, making accurate preoperative assessment essential for optimal outcomes. Furthermore, MDCT can be used to assess coronary ostial heights, sinus of Valsalva diameter, aortic root morphology, and calcium distribution patterns, all of which influence valve deployment feasibility and potential complications [18]. Given its predictive utility, routine preoperative MDCT is increasingly considered the standard of care for all patients undergoing valve replacement, especially those with small annuli (e.g., anticipated <23 mm bioprosthesis) or other high-risk features. This approach allows the multidisciplinary heart team to anticipate and prevent PPM rather than manage it retrospectively.

Following valve implantation, postoperative evaluation of PPM is primarily conducted through transthoracic echocardiography, which remains the diagnostic gold standard for assessing prosthetic valve hemodynamics [19,20]. A comprehensive echocardiographic assessment provides both immediate postoperative baseline values and enables longitudinal monitoring of prosthetic valve performance [21].

The combination of MDCT for preoperative prediction and echocardiography for postoperative confirmation establishes a comprehensive, evidence-based framework for PPM evaluation throughout the patient care continuum. This systematic approach represents a fundamental advancement in valve replacement care, shifting the clinical paradigm from reactive management to predictive, personalized intervention strategies.

## 5. Valve Selection and PPM Outcomes

The selection of an appropriate prosthetic valve should be individualized for each patient, incorporating considerations of age, anticipated valve durability, comorbid conditions, and risk of prosthesis–patient mismatch. The choice between mechanical and bioprosthetic valves has traditionally been guided by patient age and anticoagulation tolerance, but emerging evidence suggests that PPM risk should also factor prominently in this decision-making process [22].

Contemporary evidence challenges traditional age-based valve selection paradigms, particularly in elderly populations. A comprehensive propensity-matched analysis by Okamoto et al. investigated early and late outcomes following aortic valve replacement using bioprosthetic versus mechanical valves in 104 patients aged 75 years and older [22]. The study demonstrated comparable 30-day mortality rates between valve types (1.9% for bioprosthetic vs. 5.8% for mechanical, *p* = 0.618) and similar long-term survival at eight years (72.8% vs. 73.3%, respectively; *p* = 0.473) [22]. Valve-related complications, including thromboembolism, bleeding, and reoperation, were low and similar in both cohorts [22]. Importantly, the analysis revealed no significant differences in PPM prevalence at 10 days after AVR and at long-term follow-up of 52 months between valve types. Moderate PPM occurred in 17 patients (32.7%) receiving bioprosthetic valves compared to 13 patients (25%) with mechanical valves (*p* = 0.387). Severe PPM was observed in 4 patients (7.7%) with bioprosthetic valves versus 1 patient (2%) with mechanical valves (*p* = 0.363) [22].

Sutureless and rapid-deployment valves have demonstrated favorable hemodynamic performance with consistently low PPM rates, particularly in elderly patients and those with small annuli. In the prospective CAVALIER trial of 658 patients receiving the Perceval sutureless valve, early results demonstrated favorable hemodynamic outcomes with mean gradients of 11.6 ± 5.4 mmHg at discharge, decreasing to 10.3 ± 4.9 mmHg at 1 year, with corresponding iEOA values of 0.85 ± 0.23 cm^2^/m^2^ [23]. Similarly, the Edwards Intuity Elite rapid-deployment valve demonstrated favorable hemodynamic performance in the TRITON trial of 287 patients, with mean gradients of 8.4 ± 3.4 mmHg and EOA of 1.7 ± 0.2 cm^2^ at 1 year, and severe PPM occurring in 7.8% at 5-year follow-up [24]. These data suggest that rapid-deployment technologies may offer hemodynamic advantages without compromising effective orifice area, making them particularly attractive options for patients at higher risk for PPM.

These findings suggest that valve-specific hemodynamic characteristics may be more influential than valve type in determining PPM risk, supporting individualized selection based on patient-specific anatomical and physiological factors.

## 6. Prevalence of PPM Following Bioprosthetic Aortic Valve Implantation

Contemporary bovine pericardial valves have demonstrated superior hemodynamic performance and reduced PPM rates compared to earlier generation prostheses. Especially, the Perimount Magna and Magna Ease valves have established excellent clinical performance with particularly low PPM rates at average follow-up times of 2 and 6 years [25,26]. In a cohort of 282 patients stratified by PPM risk factors, no cases of severe PPM were observed at a mean follow-up of 5 years across all valve sizes, with indexed effective orifice areas consistently maintained above 0.65 cm^2^/m^2^ despite varying body surface area to annular diameter ratios [27]. These valves demonstrated exceptional long-term durability with 5-year survival rates exceeding 92% and no reported structural valve deterioration [28]. Long-term comparative data further support the hemodynamic advantages of bovine pericardial designs. The Perimount Magna demonstrated significantly lower 5-year PPM rates compared to the Mosaic valve (4% vs. 32.5%) and superior performance relative to the Hancock II valve (30% vs. 52%, *p* = 0.02) [29,30]. Stented porcine valves generally exhibit higher PPM rates compared to bovine pericardial alternatives, particularly in smaller sizes [31]. Comparative analysis of the CE Perimount and Medtronic Mosaic valves revealed higher severe PPM rates 10 months after AVR with the Mosaic, especially prominent in smaller valve sizes: 46.2% versus 40.0% for 21 mm valves and 22.7% versus 13% for 23 mm valves [32]. The Epic Supra valve, despite improved hemodynamics relative to conventional porcine valves through its supra-annular design, still demonstrated a higher severe PPM rate (26.8%) after 3 years [33]. These findings underscore the hemodynamic limitations inherent to porcine tissue valves, particularly in patients with smaller aortic annuli.

Stentless valves have demonstrated superior hemodynamic performance and lower PPM rates across multiple studies, though optimal valve selection must balance these hemodynamic advantages against operative complexity, surgeon experience, durability expectations, and future coronary access considerations [32,34]. In a large cohort of 345 patients, the Freedom Solo and Solo Smart valves combined showed a 13.7% overall PPM rate with only one case of severe PPM at a long-term follow-up of more than 10 years [35]. The Toronto SPV demonstrated exceptional long-term performance with no cases of PPM in direct comparison to the Mosiac valve and an overall PPM rate of 10.9% at 15-year follow-up in 515 patients [36]. In another study, 10 patients receiving Medtronic Freestyle valve reported similarly low PPM rates (2 patients) at 1-year follow-up, highlighting the consistent benefits of stentless valve design [37].

The superior hemodynamic performance of stentless valves stems from their ability to utilize the native aortic root more efficiently, eliminating the flow limitations imposed by rigid stent structures [38]. This design advantage is particularly beneficial in patients with small aortic annuli, where even modest improvements in effective orifice area can significantly impact hemodynamic outcomes and long-term survival. These results are summarized in Table 1.

Early data on next-generation bioprostheses demonstrate promising hemodynamic profiles with low PPM rates. The Inspiris Resilia valve has shown excellent early performance in a 1-year series of 487 patients, with severe PPM occurring in only 1.4% and moderate PPM in 6.2%, accompanied by substantially reduced transvalvular gradients (mean approximately 9 mmHg at 1 year) and well-preserved iEOA values [46]. In a two-center comparative study of Inspiris versus Avalus valves (n = 74 each), mid-term EOAs were comparable (1.5 vs. 1.4 cm^2^) with corresponding iEOA measurements of 0.8 vs. 0.7 cm^2^/m^2^ [47]. However, separate hemodynamic evaluation of the Avalus valve in a 48-patient cohort revealed higher PPM rates at mid-term follow-up (29% moderate, 15% severe), consistent with its slightly smaller indexed orifice area compared to contemporary bovine pericardial valves [48].

While these findings highlight the generally favorable hemodynamics of bioprosthetic and modern tissue valves, they also underscore important differences between specific valve models and emphasize the critical need for longer-term follow-up data to fully characterize PPM risk and durability outcomes.

## 7. Prevalence of PPM Following Mechanical Aortic Valve Implantation

The evolution of mechanical valve technology has yielded significant improvements in hemodynamic performance and complication rates. Modern mechanical valves predominantly feature bileaflet designs optimized for specific clinical applications. The Sorin Slimline valve incorporates supra-annular implantation capabilities with increased geometric orifice area, while the St. Jude Medical Regent valve is specifically optimized for small aortic annuli with enhanced valve area characteristics [43,49,50].

Advanced design features include the Carbomedics valve’s titanium stiffening ring to prevent leaflet escape and the ATS bileaflet valve’s low-profile pyrolytic carbon leaflets with open pivots for enhanced flow dynamics [44,45]. These technological advances have contributed to improved hemodynamic performance across the mechanical valve spectrum.

Contemporary studies demonstrate that mechanical valve PPM rates range from 8 to 16%, with higher prevalence observed in smaller valve sizes [44]. In a comprehensive analysis of 655 patients undergoing surgical aortic valve replacement with various mechanical valves, the overall PPM prevalence was 8.7% 12 months after AVR [51].

While PPM did not emerge as an independent predictor of early complications or mortality, it was associated with reduced long-term survival in higher-risk patient subgroups, including older patients, those undergoing concomitant coronary artery bypass grafting, and patients with NYHA class III/IV symptoms or left ventricular dysfunction [51].

The availability of 17 mm mechanical valves addresses a critical clinical need in patients with extremely small aortic annuli, where bioprosthetic options are typically unavailable [52]. However, these ultra-small valves carry substantial PPM risk, with moderate PPM occurring in up to 43.4% of patients by 12 months after AVR [52,53]. This high mismatch rate particularly impacts patients with pre-existing left ventricular dysfunction, contributing to increased mortality and adverse long-term outcomes.

Therefore, the selection of optimal prosthetic valves requires integration of patient-specific factors, anticipated hemodynamic performance, and long-term durability considerations. Contemporary evidence supports prioritizing hemodynamic performance through valve designs that minimize PPM risk, particularly in patients with small aortic annuli or elevated cardiac output requirements [2]. The superior performance of stentless valves and advanced bovine pericardial designs should be strongly considered in high-risk patients, while mechanical valves remain viable options when anticoagulation is acceptable and hemodynamic performance is optimized through appropriate sizing and design selection.

## 8. Comparative Outcomes Between TAVR and SAVR

Patient-prosthesis mismatch (PPM) is a critical outcome parameter in aortic valve replacement, influenced by valve type, implantation technique, and annular size. Across multiple trials, transcatheter aortic valve replacement (TAVR) has generally demonstrated lower PPM rates compared to surgical aortic valve replacement (SAVR), particularly with supra-annular self-expanding valves (SEVs) [54,55,56]. In early trials like the PARTNER trial, severe PPM occurred in 19.7% of TAVR patients versus 28.1% of SAVR patients at the first postoperative echogram, suggesting an early hemodynamic advantage for TAVR [57]. Similarly, the CoreValve US High Risk Trial reported severe PPM in 6.2% of TAVR (SEV) patients versus 25.7% with SAVR at 1 year, reinforcing the benefit of the supra-annular design [58].

Large-scale registry studies have corroborated findings from randomized trials while providing broader population-based insights. A comprehensive analysis encompassing 62,125 TAVR patients revealed moderate PPM in 24.6% and severe PPM in 12.1% of recipients at 1 year [59]. While demonstrating that PPM remains a clinically relevant issue in TAVR, these rates generally compare favorably to historical SAVR outcomes, particularly when considering the higher-risk profile of early TAVR populations.

The extension of TAVR to low-risk populations has revealed important platform-specific differences in PPM rates. The PARTNER 3 trial, evaluating balloon-expandable valve (BEV) technology in low-risk patients, demonstrated outcomes that challenged the universal superiority of TAVR for PPM prevention [54]. Moderate PPM occurred more frequently with BEV-TAVR (29.8%) compared to SAVR (23.3%), though severe PPM remained lower in the TAVR group (4.3% vs. 6.3%) at 30 days. These findings highlighted that PPM outcomes in TAVR are not uniform across all valve platforms and risk categories [54,60]. However, the Evolut Low-Risk Trial, utilizing supra-annular SEV technology, demonstrated markedly superior PPM outcomes [60]. The trial reported significantly lower rates of both moderate (5.0% vs. 15.7%) and severe PPM (1.8% vs. 8.2%) compared to SAVR at 12 months [60]. These findings reinforced the importance of valve design characteristics, particularly supra-annular positioning, in achieving favorable hemodynamic outcomes and emphasized the platform-dependent nature of PPM risk in contemporary practice.

Patients with small aortic annuli (SAA) represent a particularly challenging population for PPM prevention, as limited annular dimensions constrain the size of implantable prostheses. The SMART trial provided direct comparative evidence between TAVR platforms in this high-risk population, demonstrating significantly lower combined moderate or severe PPM rates with SEVs (11.2%) compared to BEVs (35.3%) at 30 days [55]. This substantial difference underscores the importance of platform selection in anatomically challenging cases. The VIVA trial specifically examined TAVR versus SAVR outcomes in SAA patients, revealing numerically lower severe PPM rates with TAVR (5.6%) compared to SAVR (10.3%) at 60 days, though statistical significance was not achieved [61]. Results are summarized in Table 2.

The evolving evidence base demonstrates that TAVR, particularly with supra-annular SEV technology, generally achieves lower PPM rates compared to SAVR across diverse patient populations. However, the platform-dependent nature of these outcomes necessitates individualized treatment strategies that account for patient anatomy, valve characteristics, and anticipated PPM risk.

## 9. Clinical Impact of PPM

While patient-prosthesis mismatch (PPM) was initially considered a technical consideration in valve selection, mounting evidence has established its profound impact on both immediate and long-term clinical outcomes following aortic valve replacement (AVR) [4]. The hemodynamic consequences of PPM affect cardiac remodeling, coronary physiology, and ultimately patient survival [4].

One of the most well-characterized consequences of PPM involves its detrimental effect on left ventricular (LV) remodeling following AVR [62]. The relationship between valve hemodynamics and reverse remodeling has been extensively studied, with indexed effective orifice area (iEOA) emerging as a critical determinant of LV mass regression [62]. Del Rizzo and colleagues, in a comprehensive analysis of 1103 patients receiving porcine bioprosthetic valves in the aortic position, demonstrated a strong correlation between iEOA and the degree of LV mass regression achieved post-operatively [63]. Further evidence from Tasca et al. confirmed that normalization of LV mass is negatively and independently influenced by the presence of PPM, though considerable variability exists depending on the achieved post-operative valve effective orifice area [64,65]. Importantly, these studies revealed that while some degree of LV mass regression occurs even in patients with PPM, the magnitude of improvement is substantially diminished, particularly when the increase in effective orifice area is minimal [65]. This finding has significant implications for long-term cardiac function and patient outcomes. The relationship between iEOA and transvalvular gradients follows a curvilinear pattern, suggesting that even modest improvements in iEOA can yield disproportionately beneficial effects on hemodynamic parameters. This non-linear relationship highlights the importance of maximizing effective orifice area whenever possible, as small incremental improvements may translate into substantial clinical benefits.

Beyond its effects on cardiac structure, PPM significantly impacts coronary physiology, particularly coronary flow reserve (CFR). Patients with aortic stenosis frequently experience angina despite having angiographically normal epicardial coronary arteries, primarily due to impaired CFR secondary to myocardial hypertrophy and elevated intracavitary pressures [66,67]. Rajappan and colleagues demonstrated that CFR impairment correlates more closely with valve severity and post-operative effective orifice area changes than with absolute LV mass measurements [68]. This finding has important clinical implications, as persistent PPM may significantly attenuate CFR improvement following AVR, potentially perpetuating myocardial ischemia and contributing to ongoing LV dysfunction. The persistence of abnormal coronary physiology in the setting of PPM may also explain why some patients continue to experience anginal symptoms despite technically successful valve replacement [69].

The hemodynamic consequences of PPM translate into measurable impacts on early clinical outcomes. PPM has been consistently associated with lower postoperative cardiac index and higher rates of heart failure, with effects that demonstrate clear severity dependence [70]. Rao et al., in a series of 2154 patients, reported significantly higher 30-day mortality rates in patients with PPM compared to those without (7.9% vs. 4.6%, *p* = 0.03), though PPM did not emerge as an independent predictor of early death in their multivariate analysis [71]. However, other investigators have demonstrated more profound early mortality effects. In a cohort of 1265 AVR patients, the presence of moderate PPM increased in-hospital mortality risk 2.1-fold, while severe PPM was associated with an 11.4-fold increase in mortality risk [72]. These effects were particularly pronounced in patients with reduced left ventricular ejection fraction (≤40%), where mortality reached 67% in those with severe PPM, highlighting the synergistic detrimental effects of PPM and pre-existing ventricular dysfunction [72].

The clinical consequences of PPM extend well beyond the perioperative period, with multiple studies demonstrating significant impacts on long-term survival. Rao and colleagues, in their analysis of 2516 patients with bioprosthetic valves, found that 12-year survival was substantially lower in patients with severe PPM (iEOA ≤ 0.75 cm^2^/m^2^) compared to those with adequate valve sizing (75.5% vs. 84.2%, *p* = 0.004) [71]. This survival difference persisted after adjustment for relevant covariates, establishing PPM as an independent predictor of late mortality [72]. Complementary evidence from Tasca et al. demonstrated significantly reduced 5-year survival (82% vs. 93%) and cardiac event-free survival (75% vs. 87%) in patients with PPM compared to those without mismatch [73]. Perhaps most compellingly, Mohty-Echahidi and colleagues reported 8-year survival of only 41% in patients with severe PPM, compared to 65% for moderate PPM and 74% for those without mismatch, reinforcing the severity-dependent nature of PPM’s impact on long-term outcomes [74].

An often-overlooked consequence of PPM involves its impact on hemostatic function. Vincentelli et al. demonstrated that von Willebrand factor abnormalities, commonly seen in severe aortic stenosis due to high shear stress conditions, may persist in patients with PPM following AVR [75]. The continued presence of elevated transvalvular gradients maintains the high shear stress environment that disrupts von Willebrand factor multimers, potentially explaining the increased bleeding complications observed in some PPM patients [75]. These hemostatic abnormalities represent an important area for future investigation, as they may contribute to both bleeding complications and potentially thrombotic events through complex interactions between shear stress, platelet function, and coagulation factors.

Overall, the clinical consequences of PPM span from reduced LV remodeling and persistent ischemia to increased rates of heart failure, bleeding, and both early and late mortality. These data support routine efforts to avoid PPM, especially in patients with small annuli or impaired LV function, where its impact is most profound.

## 10. Reintervention Strategies After SAVR

**Figure 1:**
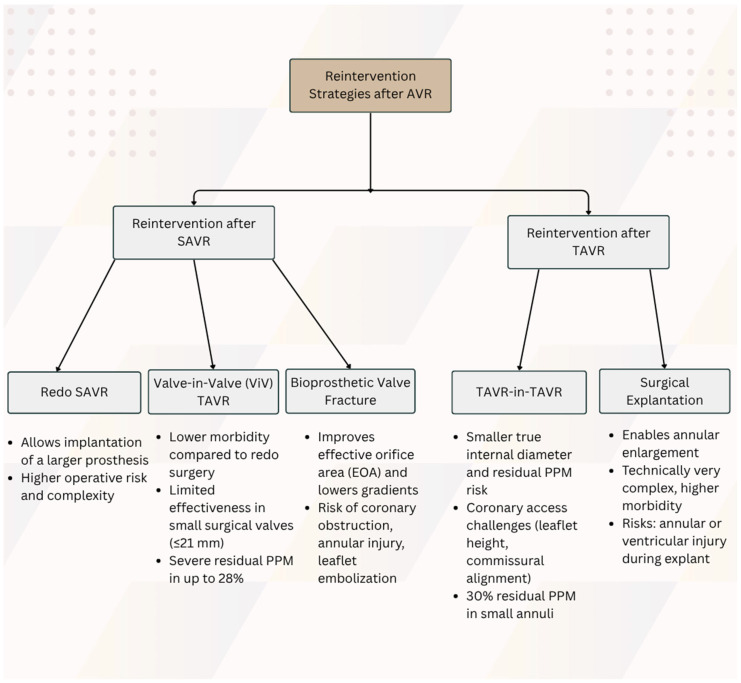
Summary of reintervention strategies after AVR.

PPM has emerged as a critical determinant of long-term outcomes following aortic valve replacement, with its clinical significance extending far beyond hemodynamic considerations. The classification of PPM severity, moderate (indexed effective orifice area [iEOA] 0.65–0.85 cm^2^/m^2^) and severe (iEOA ≤ 0.65 cm^2^/m^2^), correlates directly with adverse clinical outcomes, including persistent transvalvular gradients, impaired regression of left ventricular hypertrophy, and increased mortality risk [70]. The clinical imperative for reintervention is underscored by compelling outcome data demonstrating that patients with iEOA ≤ 0.80 cm^2^/m^2^ experience a 60% increased risk of postoperative heart failure, as reported by Ruel and colleagues [76]. These statistics highlight the urgent clinical need for timely and effective re-intervention strategies in appropriately selected patients, necessitating a comprehensive understanding of available therapeutic approaches and their relative merits in different clinical scenarios (Figure 1).

For patients initially treated with surgical aortic valve replacement (SAVR) who develop clinically significant PPM, re-intervention options encompass both surgical and transcatheter modalities, each with distinct advantages and limitations. Redo SAVR, while associated with increased procedural complexity and morbidity compared to primary operations, offers the unique advantage of enabling annular enlargement techniques. These procedures, including the well-established Manouguian and Nicks procedures, allow for accommodation of larger prostheses by surgically expanding the aortic annulus [77,78]. The Manouguian technique involves enlargement of the aortic annulus through the non-coronary sinus, extending the incision into the anterior leaflet of the mitral valve, while the Nicks procedure achieves enlargement through the non-coronary and right coronary commissure [77,78]. More recently, the Yang technique, utilizing a Y-shaped incision and rectangular patch augmentation, has been introduced as a versatile alternative that can achieve greater annular expansion with less disruption of adjacent structures [79]. These approaches can increase the annular diameter by 2–4 mm, potentially allowing implantation of a prosthesis one or two sizes larger than would otherwise be possible. The choice among these techniques often depends on patient anatomy, surgical experience, and the extent of enlargement required. However, these procedures also require considerable surgical expertise and are associated with increased operative time, bleeding risk, and potential for complications related to mitral valve injury or conduction system damage.

Valve-in-valve (ViV) TAVR has gained increasing acceptance as the preferred approach for high-risk patients requiring re-intervention after failed SAVR, offering reduced procedural morbidity compared to surgical reoperation [80]. However, the hemodynamic outcomes of ViV TAVR are significantly influenced by the characteristics of the underlying surgical prosthesis, particularly its size and design. The ViVID registry, which represents the largest compilation of ViV TAVR experience, has provided crucial insights into the limitations of this approach in patients with small surgical valves [81]. Among patients with prior SAVR undergoing ViV TAVR, those with small surgical valves (≤21 mm) demonstrated substantially higher rates of severe residual PPM, reaching up to 28% in some series [82]. This finding reflects the inherent geometric constraints imposed by small surgical valve frames, which limit the effective internal diameter available for transcatheter valve expansion. The clinical implications of this limitation are particularly relevant given that patients receiving small surgical valves often have small native annuli, making them inherently prone to PPM-related complications.

Bioprosthetic valve fracture (BVF) has emerged as a transformative adjunctive technique that significantly expands the therapeutic potential of ViV TAVR, particularly in patients with small surgical valves. This technique involves the deliberate fracture of the surgical valve frame using high-pressure balloon inflation, thereby eliminating the geometric constraints imposed by the original prosthesis and allowing for appropriate expansion of the transcatheter valve [83]. The hemodynamic benefits of BVF are substantial, with studies demonstrating significant improvements in effective orifice area and reductions in transvalvular gradients compared to standard ViV TAVR [83].

The technique has proven particularly valuable in patients with small, rigid surgical valve frames where conventional ViV TAVR would be expected to result in severe residual PPM [80]. However, BVF requires careful patient selection and procedural planning, as the technique carries specific risks including coronary obstruction, annular injury, and potential for leaflet embolization [84].

## 11. Reintervention Strategies After TAVR

Patients initially treated with TAVR who subsequently develop valve failure or PPM-related complications present unique challenges for re-intervention (Figure 1). TAVR-in-TAVR procedures, while technically feasible, are complicated by several factors that distinguish them from ViV TAVR in surgical valves. The true internal diameter of transcatheter valves, particularly balloon-expandable platforms, may be smaller than their nominal size, potentially limiting the effective orifice area achievable with a second transcatheter valve [85]. Coronary access represents another critical consideration in TAVR-in-TAVR procedures, as the combination of two transcatheter valve frames may significantly compromise future coronary interventions [85]. The leaflet height and commissural alignment of the original TAVR prosthesis can create geometric challenges for coronary cannulation, making careful procedural planning essential. Additionally, the lack of established fracture techniques for transcatheter valve frames limits options for optimizing the internal geometry of the original prosthesis.

Clinical experience with TAVR-in-TAVR has revealed concerning rates of residual PPM, particularly in patients with small native annuli. Barbanti and colleagues reported that TAVR-in-TAVR procedures in small annuli resulted in iEOAs < 0.85 cm^2^/m^2^ in over 30% of cases, suggesting a substantial risk of recurrent or persistent PPM [86]. These findings highlight the importance of careful patient selection and the potential need for alternative strategies in patients at high risk for hemodynamically significant residual stenosis.

In cases where transcatheter re-intervention is not feasible or has failed to achieve acceptable hemodynamic results, surgical explantation of the original TAVR prosthesis may be necessary. This approach allows for annular enlargement techniques similar to those used in conventional surgical re-operations, but is associated with significantly increased operative complexity and morbidity [87]. The presence of the transcatheter valve frame and associated calcification can make surgical planes more difficult to identify, increasing the risk of annular or ventricular injury during explantation [87].

Given the prognostic implications of PPM, particularly in patients with poor LV function or elevated preoperative gradients, tailored re-intervention strategies are essential. Severe PPM should prompt consideration of BVF during ViV TAVR or complete surgical redo with annular enlargement if feasible. Imaging-guided procedural planning, use of supra-annular TAVR devices, and selection of low-profile, high-performance prostheses are strategies that may optimize re-intervention outcomes. Ongoing innovations aim to minimize the risk of residual PPM and improve hemodynamics, with a growing role for CT-based annular assessment and computational modeling to personalize valve selection and intervention.

## 12. Conclusions

Despite substantial progress in understanding and preventing PPM, important knowledge gaps remain that warrant further investigation. First, improved risk stratification through integration of multimodality imaging with machine learning algorithms may enable more precise prediction of individual PPM risk and patient-specific hemodynamic outcomes. Second, the long-term impact of PPM in younger, lower-risk patients undergoing TAVR requires dedicated study, as existing data predominantly reflect older, higher-risk cohorts with shorter follow-up. Third, optimal iEOA thresholds require validation in specific subgroups, including obese patients with small annuli who may tolerate lower iEOA differently, and those with reduced left ventricular function or heart failure with preserved ejection fraction, where the hemodynamic consequences of PPM may be particularly detrimental. Fourth, the development of novel prosthetic designs that maximize effective orifice area while minimizing complications such as conduction disturbances and paravalvular leak remains a priority. Finally, as TAVR-in-TAVR and ViV procedures become increasingly common, strategies to prevent PPM during index TAVR require systematic investigation, including the role of intentional annular sizing to accommodate future valve implantation.

In conclusion, prosthesis–patient mismatch (PPM) is a common and modifiable complication of aortic valve replacement that occurs when the prosthetic valve is small relative to the patient’s body surface area, leading to elevated transvalvular gradients and impaired hemodynamics. Severe PPM has been consistently associated with worse postoperative outcomes, including less regression of left ventricular hypertrophy, increased adverse cardiac events, and reduced survival. Accurate annular sizing, indexing the expected effective orifice area (EOA) to body surface area, and considering surgical annular enlargement are critical preventive strategies. As treatment strategies evolve, particularly with the increased use of TAVR in younger, lower-risk patients, avoiding PPM at the time of initial intervention becomes essential to optimize long-term outcomes and allow for future re-intervention.

## Figures and Tables

**Table 1:** Summary of PPM prevalence in bioprosthetic and mechanical valves.

Valve Type	Valve Name	Size (mm)	Study Outcomes
Stented Pericardial	Carpentier-Edwards Perimount	19–27	Excellent durability, low gradients [26,27].
	Mitroflow	19–27	Small EOA in smaller sizes; early SVD noted in some reports [39,40].
	Avalus	19–27	Newer valve with favorable early outcomes [41].
Stent-less Pericardial	Freedom Solo Smart	21–27	Excellent EOA in all sizes; effective in small annuli [35].
Stented Porcine	Carpentier-Edwards Supra-Annular	19–27	Reliable performance; lower EOA than pericardial valves [32].
	Mosaic Valve	19–27	Good durability; moderate gradients in smaller sizes [36].
	Hancock II	19–27	Long-term data; hemodynamics less favorable than newer valves [30].
	Epic Valve	19–27	Improved EOA over Hancock; supra-annular profile [33].
Stent-less Porcine	Toronto SPV	21–27	High EOA; effective in small annuli [36].
Mechanical	Starr-Edwards Aortic Ball Valve	Historical	Obsolete; high gradients [42].
	Björk–Shiley Tilting Disk Valve	Historical	Known complications; obsolete [42]
	Kay-Shiley Non-tilting Disk Valve	Historical	Obsolete [42]
	Sorin Bicarbon Slimline	19–27	Favorable hemodynamics; compact design [43].
	St. Jude Medical Regent	19–27	Supra-annular design; high EOA [43,44].
	CarboMedics Mechanical Valve	19–27	Durable; less favorable EOA in smaller sizes [44].
	Medtronic Open Pivot	19–27	Low thrombogenicity; reliable gradients [32]
	On-X Aortic Valve	19–27	High EOA; allows low INR; strong hemodynamic performance [44,45].
	ATS Mechanical Valve	19–27	Favorable profile; low gradients [44,45].

**Table 2:** Summary of PPM Prevalence in Major TAVR vs. SAVR Comparative Trials.

Trial	Population	Valve Platform	Moderate PPM	Severe PPM	Key Finding	Time Point
PARTNER	High-risk patients with severe AS	TAVR: BEV (SAPIEN) SAVR: Various surgical valves	Not separately reported	TAVR: 19.7% SAVR: 28.1%	Early hemodynamic advantage for TAVR in high-risk patients	First postoperative echo
CoreValve US High Risk	High-risk patients with severe AS	TAVR: SEV (CoreValve) SAVR: Various surgical valves	Not separately reported	TAVR: 6.2% SAVR: 25.7%	Supra-annular SEV design demonstrates substantial reduction in severe PPM	1 year
TAVR Registry Study	Broad TAVR population (62,125 patients)	Various TAVR platforms	24.6%	12.1%	PPM remains clinically relevant in TAVR despite lower rates than historical SAVR	1 year
PARTNER 3	Low-risk patients with severe AS	TAVR: BEV (SAPIEN 3) SAVR: Various surgical valves	TAVR: 29.8% SAVR: 23.3%	TAVR: 4.3% SAVR: 6.3%	BEV-TAVR may have higher moderate PPM than SAVR in low-risk patients; valve platform matters	30 days
Evolut Low Risk	Low-risk patients with severe AS	TAVR: SEV (Evolut) SAVR: Various surgical valves	TAVR: 5.0% SAVR: 15.7%	TAVR: 1.8% SAVR: 8.2%	Supra-annular SEV technology achieves markedly superior PPM outcomes compared to SAVR in low-risk population	12 months
SMART	Small aortic annulus (<430 mm^2^ by CT)	TAVR: SEV vs. BEV comparison	Combined moderate/severe: SEV: 11.2% BEV: 35.3%	Not separately reported	SEV substantially outperforms BEV in small annuli; platform selection critical in anatomically challenging cases	30 days
VIVA	Small aortic annulus	TAVR: Various platforms SAVR: Various surgical valves	Not separately reported	TAVR: 5.6% SAVR: 10.3%	Numerically lower severe PPM with TAVR vs. SAVR in small annuli (not statistically significant)	60 days
